# Position in Labor

**Published:** 1874-01

**Authors:** 


POSITION IN LABOR.—Dr. William B. Williams, of Mississippi, in a private letter to us, says: "By acting on the principles there laid down, (referring to Dr. Smith's article on "Position in Labor," published in the October number of the RECORD), I was enabled recently to terminate a case of lingering labor satisfactory to both mother and child, in twenty minutes, or perhaps in less time. An old midwife had been in attendance some two days, and a physician from early in the morning until two o'clock P.M., when I was called and requested to take instruments along with me. The doctor told me on my arrival the case was a very bad one, and could not be relieved without the use of instruments. Upon examination I thought differently, and suggested a change in the mother's position, to which he consented, at the same time expressing a doubt as to success. The change was made and delivery effected in twenty minutes."

The editors thank Dr. W. for the interest he manifests in the RECORD, and for the subscriptions sent.

